# The Complexity Entropy Analysis of a Supply Chain System Considering Recovery Rate and Channel Service

**DOI:** 10.3390/e21070659

**Published:** 2019-07-04

**Authors:** Qiuxiang Li, Mengnan Shi, Qing Deng, Yi-min Huang

**Affiliations:** 1 Institute of Modern Logistics, Henan University, Kaifeng 475004, China; 2 Institute of Management Science and Engineering, Henan University, Kaifeng 475004, China; 3 School of Business, Henan University, Kaifeng 475004, China; 4 School of Management & Economics, North China University of Water Resources and Electric Power, Zhengzhou 450046, China

**Keywords:** closed-loop supply chain, recovery rate, service, entropy

## Abstract

In this paper, we study a dual-channel closed-loop supply chain in which a manufacturer considers the market waste products recovery and remanufacture, and a retailer considers provide services to customers. We build a Stackelberg game model and a centralized game model in a static and dynamic state, respectively, and analyze the two dynamic models by mathematical analysis and explore the stability and entropy of the two models using bifurcation, the basin of attraction, chaotic attractors, and so on. The influences of service level and profit distribution rate on the system’s profit are discussed. The theoretical results show that higher price adjustment speed will lead to the system lose stability with a larger entropy value. In the Stackelberg game model, the stability of the system increases as the service value and the recovery rate increases; in the centralized model, the stability of the system decreases with the increase of the service value and increases with the recovery rate increases. When the Stackelberg game model is in a stable state, the manufacturer’s profit increases first and then decreases, and the retailer’s profit first decreases and then increases as the service value of the retailer increases. The research will serve as good guidance for both the manufacturer and retailer in dual-channel closed-loop supply chains to improve decision making.

## 1. Introduction

With the continuous deterioration of economic globalization and enterprise competition environment, and to achieve long-term sustainable development, environmental problems become a consideration for the development of enterprises [1]. Establishing a reasonable and efficient closed-loop supply chain to collect and remanufacture waste products plays an important role in improving the environmental and economic benefits of enterprises [2,3]. Therefore, more end-of-life (EOL) products are environmentally disposed of by enterprises [4,5]. The remanufacturing of EOL products is conducive to saving natural resources, energy, and protecting the environment. Manufacturers not only sell products to customers through traditional and direct channels, but also collect and remanufacture EOL products through recycling channels to save costs [6], reduce consumption, and protect the environment. With the development of economic globalization, more and more retailers provide sales services to consumers, and sales services play an important role in the process of consumption [7,8]. The closed-loop supply chain and channel services have been studied from multiple aspects and perspectives, while few papers simultaneously considered the closed-loop supply chain and retailer’s service input in dual-channel supply chains. Therefore, this paper establishes the dual-channel closed-loop supply chain based on the product recovery rate of the manufacturer, remanufacturing cost difference, and the retailer service input, and constructs the centralized and decentralized dynamic game models by using game theory, system dynamics, and other methods. The optimal price strategy of the dynamic system and the evolutionary complexity of the dynamic price game model are analyzed by bifurcation, the basin of attraction, chaotic attractors, the Lyapunov exponent and so on [9,10]. This paper can provide some references for managers related to recycled products and provided services.

## 2. Literature Review

Many scholars have studied the closed-loop supply chain from multiple aspects and perspectives. Ismail and Theodore [11] established a closed-loop supply chain composed of multiple periods and multiple echelons. This model studies the total cost minimization of supply chain network under incomplete quality production conditions. Reimann et al. [12] studied the closed-loop supply chain in which a manufacturer performs process innovation and a manufacturer or retailer performs remanufacturing and found that decentralized supply chain is more suitable for process innovation. Yi et al. [13] established a closed-loop supply chain with dual recycling channels. In the positive channel, the manufacturer produces new products and the retailer recycle old products and remanufacture; in the reverse channel, the retailer and the third party collector simultaneously collect old products. They studied the optimal strategy for collection decisions. Zheng et al. [14] investigated a three-stage closed-loop supply chain consisting of a manufacturer, a distributor, and a retailer, in which the retailer has fair concern behaviors. They analyzed interactions between participants in both cooperative and non-cooperative situations. Guo and Ma [15] put forward a closed-loop supply chain in which the retailer recycle waste products, and discussed the influence of complex system dynamics and system parameters on the Stackelberg game. Panda et al. [16] studied a closed-loop supply chain, in which a retailer recycles old products and has a social responsibility; they analyzed the revenue sharing problem of supply chain members. 

Wei et al. [17] studied the optimal decision problem of a closed-loop supply chain and discussed how the manufacturer and retailer make decisions on wholesale price, retail price, and recycling rate under the condition of symmetric and asymmetric information. Hasanov et al. [18] studied the coordination of order quantity and remanufacturing in a four-layer closed-loop supply chain, the results show that the higher collection rates of waste products are more beneficial to the supply chain. Xie et al. [19] considered the relationship between recovery rates and studied the contract coordination problem under centralized decision and decentralized decision in double-channel closed-loop supply chains, and gave the optimal pricing of the supply chain. Dai et al. [20] analyzed the impact of delay parameters on the system, such as recovery rate of waste products, direct price, carbon quota subsidy, and carbon tax. Many scholars have also studied the optimal pricing of closed-loop supply chains under different scenarios. Wang et al. [21] used the Stackelberg model to study the optimal pricing strategies of the manufacturer, the retailer, and remanufacturer. Gan et al. [22] constructed a closed-loop supply chain-pricing model for short-life-cycle products, which is composed of a manufacturer, a retailer, and a recycler. They found that the acceptance of remanufactured products and direct channel preference affect supply chain pricing. Hong et al. [23] found that in the closed-loop supply chain, advertising costs have a great influence on channel members on the recycling decisions, pricing strategies, and profits of second-hand products. Chen and Bell [24] discussed the influences of recycling channel cost input and recycling price pricing strategy on the retailer’s pricing and ordering decisions. Under the centralized and decentralized models, He et al. [25] numerically studied the impact of customers’ intuitive impressions on different collection channels. He [26] considered the closed-loop supply chain with uncertain supply and demand, and studied the optimal decision of recovery price and remanufacturing of supply chain members in the centralized and decentralized models. 

Many scholars have studied the problem of price decision and service decision under the channel service provided by retailers and manufacturers, Zhou et al. [27] studied the two-channel supply chain provided by the retailer and analyzed the impact of free-rider behavior of supply chain members on channel pricing, profit, and service strategy under differentiated and undifferentiated schemes. Dan et al. [28] concluded that the service input of the retailer in the supply chain not only has an impact on themselves, but also has a great impact on the manufacturer. Li and Li [29] considered a two-channel supply chain, in which the retailer provides value-added services and has fair behavior concerns, they studied the impact of the retailers’ behavioral factors on supply chain pricing decisions. Jena and Sarmah [30] studied the model of two manufacturers providing services to consumers through the retailer, as well as the price decision and service decision problems of supply chain members. Zhang et al. [31] studied the optimal decision of the supply chain, in which a manufacturer and a service provider cooperate, and found that the supply chain can achieve Pareto improvement when relevant parameters are suitable for some conditions. There are also studies on the impact of service levels on dynamic systems or consumer channel choices. Dumrongsiria et al. [32] believe that price and service are the main factors for consumers when choosing a shopping channel. Service level not only influences consumers’ choice but also influences the optimal pricing of sales channels. Zhang and Wang [33] constructed a bullwhip effect model and dynamic pricing strategy model of retailers and manufacturers and analyzed the influence of service levels on the complexity of dynamic systems and bullwhip effect. There are also studies in different service contexts, under the fact that optimal inventory allocation has a significant impact on retail profits, Protopappa-Sieke et al. [34] proposed the optimal inventory allocation strategy for multiple service level contracts. Li and Ma [35] studied the influence of the manufacturer’s risk attitude on the market strategies of both participants under two market conditions of service spillover effect and non-service spillover effect. Li and Li [36] considered three strategies of the no-service, ex-ante, and ex-post service effort strategies, the influence of showrooming effect on enterprise pricing and service efforts in dual-channel supply chains are studied. Fan et al. [37] studied the pricing and service cooperation decision-making of supply chain members under BOPS mode. In the master-slave game, Sadjadi et al. [38] studied two manufacturers and a retailer who simultaneously played games under service, price discount, and contract. 

This paper is organized as follows: The Stackelberg game model is developed in Section 2. Section 3 analyses the influence of parameters on system behavior. Section 4 presents and analyses the centralized game model. The influence of parameters on system performance was studied in Section 5. The conclusions are given in Section 6.

## 3. The Stackelberg Game Model

### 3.1. Model Description

**Figure 1:**
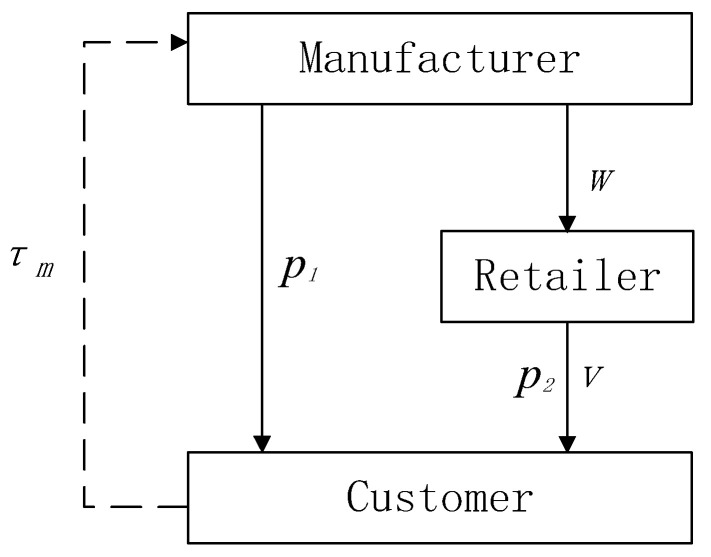
The closed-loop supply chain model.

The dual-channel closed-loop supply chain system is shown in Figure 1, in which the manufacturer sells products in direct selling channel at the direct selling price $p_{1}$ and directly recycles waste product for remanufacturing in the recycling channel, the retailer buys products from the manufacturer at the wholesale price w and sells them to customers at the retail price $p_{2}$ in the traditional channel. In a closed-loop supply chain with two channels, the manufacturer and retailer make price decisions with the goal of maximizing profits. The manufacturer takes waste recovery into account, and the retailer provides services to consumers (see Figure 1).

The model developed in this paper is based on the following assumptions:

(1) The manufacturer recycles and remanufactures old products.
(2) The unit cost of the new product is greater than that off a remanufactured product.
(3) Recycled remanufactured products and new products have the same selling price.

The symbols used in this paper and their meanings are listed in Table 1.

### 3.2. Model Construction

The demand functions of the direct channel and traditional channel are introduced as follows:

(1) $$\{\begin{array}{l} Q_{1}=\theta a-b_{1}p_{1}+k\left(p_{2}-v\right)\\ Q_{2}=\left(1-\theta \right)a-b_{2}\left(p_{2}-\nu \right)+kp_{1} \end{array}$$

Letting $\frac{\eta v^{2}}{2}$ is the unit service cost of the retailer and $\frac{\xi \tau_{m}^{2}}{2}$ is the recovery cost of the manufacturer. The cost difference between the new product and the remanufactured product is $\Delta =c_{1}-c_{2}$. The production cost of the unit product is composed of the proportion of recovery rate as follows:

$$c=\tau_{m}c_{2}+\left(1-\tau_{m}\right)c_{1}=c_{1}-\tau_{m}\Delta$$

The profit functions of the manufacturer and retailer are as follows:

(2) $$\{\begin{array}{l} \pi_{m}=\left(w-c_{1}+\tau_{m}\Delta \right)Q_{2}+\left(p_{1}-c_{1}+\tau_{m}\Delta \right)Q_{1}-\frac{\xi \tau_{m}^{2}}{2}\\ \pi_{r}\;=\left(p_{2}-w-\frac{\eta v^{2}}{2}\right)Q_{2} \end{array}$$

The total profit of the two channels is:

(3) $$\pi =\pi_{m}+\pi_{r}=\left(p_{2}-\frac{\eta v^{2}}{2}-c_{1}+\tau_{m}\Delta \right)Q_{2}+\left(p_{1}-c_{1}+\tau_{m}\Delta \right)Q_{1}-\frac{\xi \tau_{m}^{2}}{2}$$

The manufacturer and the retailer play the master-slave game, namely, the manufacturer makes the price decision first, and the retailer makes its own price decision according to the manufacturer’s decision. We first obtain the optimal price decision ($p_{2}$) of the retailer, then get w and $p_{1}$ of the manufacturer. 

Let $\frac{\partial \pi_{r}}{\partial p_{2}}=0$, we can obtain:

(4) $$p_{2}=\frac{k}{2b_{2}}p_{1}+\frac{w}{2}+A_{1}$$

where $A_{1}=\frac{\left(1-\theta \right)a}{2b_{2}}+\frac{v}{2}+\frac{\eta v^{2}}{4}$;

Then taking the first-order partial derivatives of the manufacturer’s profit function $\pi_{m}$ with respect to w and $p_{1}$:

(5) $$\{\begin{array}{l} \frac{\partial \pi_{m}}{\partial w}=kp_{1}-b_{2}w-b_{2}A_{1}+A_{2}\\ \frac{\partial \pi_{m}}{\partial p_{1}}=\left(\frac{k^{2}}{b_{2}}-2b_{1}\right)p_{1}+kw+kA_{1}+A_{3} \end{array}$$

where $A_{2}=\left(1-\theta \right)a+b_{2}v+\frac{\left(b_{2}-k\right)\left(c_{1}-\tau_{m}\Delta \right)}{2}$;

$$A_{3}=\theta a+\left(b_{1}-\frac{k^{2}}{2b_{2}}-\frac{k}{2}\right)\left(c_{1}-\tau_{m}\Delta \right)-kv$$

Then, the Hesse matrix of the manufacturer’s profit is as follows:

$$H^{D}=\left[\begin{array}{cc} -b_{2} & k\\ k & \frac{k^{2}}{b_{2}}-2b_{1} \end{array}\right]$$

Due to $-b_{2}<0,\;b_{1}>k,b_{2}>k$; then

$$\left|\begin{array}{cc} -b_{2} & k\\ k & \frac{k^{2}}{b_{2}}-2b_{1} \end{array}\right|=b_{1}b_{2}-k^{2}>0$$

The Hesse matrix is negative definite, so the manufacturer’s profit function is concave and only has the maximum solution, by solving $\frac{\partial \pi_{m}}{\partial w}=0,\frac{\partial \pi_{m}}{\partial p_{1}}=0$, we can get the optimal solution of the manufacturer:

(6) $$w^{*}=\frac{b_{1}\left(1-\theta \right)a+k\theta a}{2\left(b_{1}b_{2}-k^{2}\right)}+\frac{\left(c_{1}-\tau_{m}\Delta \right)}{2}+\frac{v}{2}-\frac{\eta v^{2}}{4}$$

(7) $$p_{1}{}^{*}=\frac{k\left(1-\theta \right)a+b_{2}\theta a}{2\left(b_{1}b_{2}-k^{2}\right)}+\frac{\left(c_{1}-\tau_{m}\Delta \right)}{2}$$

Substituting the Equations (6) and (7) into Equation (4), we can obtain:

(8) $$p_{2}{}^{*}=\frac{b_{1}\left(1-\theta \right)a+k\theta a}{2\left(b_{1}b_{2}-k^{2}\right)}+\frac{\left(1-\theta \right)a+\left(b_{2}+k\right)\left(c_{1}-\tau_{m}\Delta \right)}{4b_{2}}+\frac{3v}{4}+\frac{\eta v^{2}}{8}$$

Substituting the Equations (6)–(8) into Equations (2) and (3):

$$\{\begin{array}{l} \pi_{m}^{*}=\left(w^{*}-c_{1}+\tau_{m}\Delta \right)\left[\left(1-\theta \right)a-b_{2}\left(p_{2}{}^{*}-\nu \right)+kp_{1}{}^{*}\right]+\left(p_{1}{}^{*}-c_{1}+\tau_{m}\Delta \right)\left[\theta a-b_{1}p_{1}{}^{*}+k\left(p_{2}{}^{*}-v\right)\right]-\frac{\xi \tau_{m}^{2}}{2}\\ \pi_{r}^{*}=\left(p_{2}{}^{*}-w^{*}-\frac{\eta v^{2}}{2}\right)\left[\left(1-\theta \right)a-b_{2}\left(p_{2}{}^{*}-\nu \right)+kp_{1}{}^{*}\right]\\ \pi^{*}=\left(p_{2}{}^{*}-\frac{\eta v^{2}}{2}-c_{1}+\tau_{m}\Delta \right)\left[\left(1-\theta \right)a-b_{2}\left(p_{2}{}^{*}-\nu \right)+kp_{1}{}^{*}\right]+\left(p_{1}{}^{*}-c_{1}+\tau_{m}\Delta \right)\left[\theta a-b_{1}p_{1}{}^{*}+k\left(p_{2}{}^{*}-v\right)\right]-\frac{\xi \tau_{m}^{2}}{2} \end{array}$$

As a matter of fact, when players making price decisions, they can’t get the complete information of the whole market and adjust their price strategy with bounded rationality. Participants have always wanted to make more profits by adopting various decisions through active management behavior. Therefore, we establish a dynamic price model of the dual-channel closed-loop supply chain. The manufacturer decides the wholesale price based on the limited rationality and decides the direct selling price based on the adaptive expectation. The manufacturer makes their next period price decision based on the marginal utility of the current period. When the current marginal utility is positive, the price will be raised in the next period; otherwise, the price will be reduced in the next period. The long-term price forecasting system is as follows:

$$\{\begin{array}{l} w\left(t+1\right)\;=w\left(t\right)+\alpha_{1}w\left(t\right)\frac{\partial \pi_{m}}{\partial w}\\ p_{1}\left(t+1\right)=\alpha_{2}p_{1}\left(t\right)+\left(1-\alpha_{2}\right)p_{1}^{*} \end{array}$$

We obtain:

(9) $$\{\begin{array}{l} w\left(t+1\right)=w\left(t\right)+\alpha_{1}w\left(t\right)\;\left[kp_{1}-b_{2}w+\frac{\left(1-\theta \right)a}{2}+b_{2}\left(\frac{v}{2}-\frac{\eta v^{2}}{4}\right)+\frac{\left(b_{2}-k\right)\left(c_{1}-\tau_{m}\Delta \right)}{2}\right]\\ p_{1}\left(t+1\right)=\alpha_{2}p_{1}\left(t\right)+\left(1-\alpha_{2}\right)\;\left(\frac{k\left(1-\theta \right)a+b_{2}\theta a}{2\left(b_{1}b_{2}-k^{2}\right)}+\frac{\left(c_{1}-\tau_{m}\Delta \right)}{2}\right)\\ p_{2}\left(t\right)=\frac{1}{2}w\left(t\right)+\frac{k}{2b_{2}}p_{1}\left(t\right)+\frac{2\left(1-\theta \right)a+2vb_{2}+b_{2}\eta v^{2}}{4b_{2}} \end{array}$$

where $\alpha_{1}$ is the adjustment parameters of finite rationality and $\alpha_{2}$ is the adaptive adjustment parameters $\left(\alpha_{1}>0,\;0<\alpha_{2}<1\right)$.

In the System (9), the manufacturer adjusts the decision variables w(t) and $p_{1}\left(t\right)$ by adjusting $\alpha_{1},\alpha_{2}$, the retailer’s decision variables $p_{2}\left(t\right)$ are directly related to w(t) and $p_{1}\left(t\right)$.

### 3.3. Model Analysis

We can obtain two equilibrium solutions by making w(t+1) =w(t), $p_{1}\left(t+1\right)=\alpha_{2}p_{1}\left(t\right)+\left(1-\alpha_{2}\right)p_{1}^{*}$:

$$E_{0}=\left(0,\;\frac{k\left(1-\theta \right)a+b_{2}\theta a+2b_{2}kv}{2\left(b_{1}b_{2}-k^{2}\right)}+\frac{\left(c_{1}-\tau_{m}\Delta \right)}{2}\right);$$

$$\begin{array}{l} \;E_{1}=\left(\frac{b_{1}\left(1-\theta \right)a+k\theta a+\left(b_{1}b_{2}+k^{2}\right)v}{2\left(b_{1}b_{2}-k^{2}\right)}+\frac{\left(c_{1}-\tau_{m}\Delta \right)}{2}-\frac{\eta v^{2}}{2},\;\frac{k\left(1-\theta \right)a+b_{2}\theta a+2b_{2}kv}{2\left(b_{1}b_{2}-k^{2}\right)}+\frac{\left(c_{1}-\tau_{m}\Delta \right)}{2}\right)\\ =(\begin{array}{cc} p_{1}{}^{*}, & w^{*}) \end{array}; \end{array}$$

Thus, the retail price is:

$$p_{2}^{E_{0}}=\frac{k^{2}\left(1-\theta \right)a+kb_{2}\theta a+2b_{2}k^{2}v}{4b_{2}\left(b_{1}b_{2}-k^{2}\right)}+\frac{\left(1-\theta \right)a}{2b_{2}}+\frac{k\left(c_{1}-\tau_{m}\Delta \right)}{4b_{2}}+\frac{v}{2}+\frac{\eta v^{2}}{4}$$

$$p_{2}^{E_{1}}=\frac{b_{1}\left(1-\theta \right)a+k\theta a}{2\left(b_{1}b_{2}-k^{2}\right)}+\frac{\left(3b_{1}b_{2}+k^{2}\right)v}{4\left(b_{1}b_{2}-k^{2}\right)}+\frac{\left(1-\theta \right)a+\left(b_{2}+k\right)\left(c_{1}-\tau_{m}\Delta \right)}{4b_{2}}$$

The Jacobian matrix of System (9) is given by:

(10) $$J=\left(\begin{array}{cc} 1+\alpha_{1}\left(kp_{1}-2b_{2}w+\frac{2\left(1-\theta \right)a+b_{2}\left(2v-\eta v^{2}\right)+2\left(b_{2}-k\right)\left(c_{1}-\tau_{m}\Delta \right)}{4}\right) & \alpha_{1}kw\\ 0 & \alpha_{2} \end{array}\right)$$

The stability of equilibrium points is determined by the eigenvalues of the Jacobian matrix evaluated at the corresponding equilibrium points. We substitute $E_{0}$ and $\;E_{1}$ into the Jacobian matrix (10), we can get the following proposition.

**Proposition**: $E_{0}$*is boundary equilibrium points, $E_{1}$ is the Nash equilibrium solution.*

**Proof:** For equilibrium point $E_{0}$, the Jacobian matrix of System (9) is equal to:

$$J\left(E_{0}\right)=\left(\begin{array}{cc} \alpha_{1}\left[k\left(\frac{k\left(1-\theta \right)a+b_{2}\theta a+2b_{2}kv}{2\left(b_{1}b_{2}-k^{2}\right)}+\frac{\left(c_{1}-\tau_{m}\Delta \right)}{2}\right)+\frac{2\left(1-\theta \right)a+b_{2}\left(2v-\eta v^{2}\right)+2\left(b_{2}-k\right)\left(c_{1}-\tau_{m}\Delta \right)}{4}\right]+1 & 0\\ 0 & \alpha_{2} \end{array}\right);$$

The eigenvalues at $E_{0}$ are as follows:

$$\lambda_{1}=1+\alpha_{1}\left[k\left(\frac{k\left(1-\theta \right)a+b_{2}\theta a+2b_{2}kv}{2\left(b_{1}b_{2}-k^{2}\right)}+\frac{\left(c_{1}-\tau_{m}\Delta \right)}{2}\right)+\frac{2\left(1-\theta \right)a+b_{2}\left(2v-\eta v^{2}\right)+2\left(b_{2}-k\right)\left(c_{1}-\tau_{m}\Delta \right)}{4}\right];$$

$\lambda_{2}=\alpha_{2}$.

We can see that $\lambda_{1}>1,\lambda_{2}<1$ which indicates $E_{0}$ is an unstable point. More precisely, $E_{0}$ is a saddle point for $\left|\lambda_{2}\right|<1$, which is the boundary equilibrium point. We can prove $E_{1}$ is the Nash equilibrium solution of System (9).

Next, we analyze the stability of the equilibrium point $E_{1}$.

$$J\left(E_{1}\right)=\left(\begin{array}{cc} 1+\alpha_{1}B_{1} & \alpha_{1}kw^{*}\\ 0 & \alpha_{2} \end{array}\right)$$

where $B_{1}=kp_{1}{}^{*}-2b_{2}w^{*}+\frac{2\left(1-\theta \right)a+b_{2}\left(2v-\eta v^{2}\right)+2\left(b_{2}-k\right)\left(c_{1}-\tau_{m}\Delta \right)}{4}$.

The corresponding characteristic polynomial of the System (9) in the Nash equilibrium solution can be written as follows:

$$f\left(\lambda \right)=\lambda^{2}+U_{0}\lambda +U_{1}$$

where $U_{0}=1+\alpha_{1}B_{1}+\alpha_{2};\;U_{1}=\alpha_{2}\left(1+\alpha_{1}B_{1}\right)$; $U_{0}\;\mathrm{and}\;U_{1}$ represent the trace and determinant of the Jacobian matrix J($E_{1})$, respectively.

According to the Jury’s stability condition of the equilibrium point, the necessary and sufficient conditions for obtaining the local stability of the Nash equilibrium point $E_{1}$ are:

(11) $$\{\begin{array}{l} \left(i\right):1+U_{0}+U_{1}>0\\ \left(ii\right):1-U_{0}+U_{1}>0\\ \left(iii\right):1-\left|U_{1}\right|>0 \end{array}$$

Solving the inequality Equation (11), we can obtain the stable region of the System (9). In the stable region, the System (9) is locally stable with the initial values of prices within a certain range. Because these limitations are very complex, solving the inequality Equation (11) is very complicated. Next, we give the stable region of the System (9) through numerical simulation.

## 4. Numerical Simulations

In this section, numerical simulations are used to show the dynamic behaviors and features of the System (9). We choose some parameters values as follows: $a=200,\;\theta =0.4,b_{1}=4,b_{2}=5,k=1,\eta =0.8,v=5,c_{1}=15,c_{2}=4,\Delta =c_{1}-c_{2}=11,\tau_{m}=0.2,\xi =10$.

### 4.1. The Influence of $v\;and\;\tau_{m}$ on the Stability Region of the System (9)

When the initial values are in the stability region of the Nash equilibrium, the prices of players will be fixed at the Nash equilibrium point after a series of iterations.

**Figure 2:**
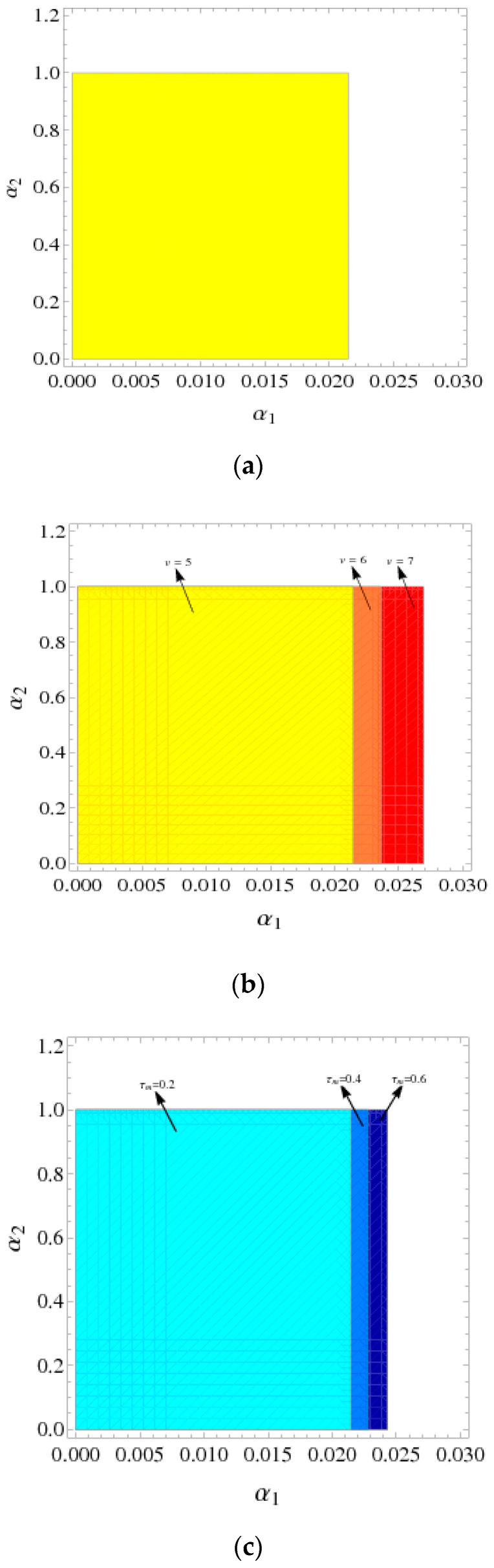
The stability regions with v and $\tau_{m}$ that have different values. (**a**) The stability regions with $v=5,\tau_{m}=0.2$; (**b**) the stability regions with different values of v; (**c**) the stability regions with different values of $\tau_{m}$.

Figure 2a shows the stability regions of the System (9) with $=5,\tau_{m}=0.2$, and the price adjustment parameter $\alpha_{1}\in \left(0,\;0.0215\right).$

In Figure 2b, when v=5,6,7, the stability regions of the System (9) are indicated by yellow, orange, and red, respectively. We can see that the stability region expands with v increases, the range of $\alpha_{1}$ increases and the range of $\alpha_{2}$ unchanged. When v=5, the range of price adjustment parameter is $\;0\;<\alpha_{1}<0.0215$; when v=6, the range of price adjustment parameters is $\;0<\alpha_{1}<0.0238$; when v=7, the range of price adjustment parameter is $0<\alpha_{1}<0.0276$. 

Similarly, Figure 2c shows that the stability regions of the System (9) expand with $\tau_{m}$ increases, the stability regions are indicated by lake blue, sky blue, and dark blue, respectively, namely, the range of $\alpha_{1}$ increases and the range of $\alpha_{2}$ unchanged. When $\tau_{m}=0.2$, the range of the price adjustment parameter is $0\;<\alpha_{1}<0.0215$; the range of price adjustment parameter is $0\;<\alpha_{1}<0.0229$ when $\tau_{m}=0.4$; when $\tau_{m}=0.6$, the range of price adjustment parameter is $0\;<\alpha_{1}<0.0243$.

From the above analysis, as the manufacturer recovery rate $\tau_{m}$ and retailer service value v increases, the System is more stable and the manufacturer and retailer have more space for price decisions, which also means more competition in the market.

**Figure 3:**
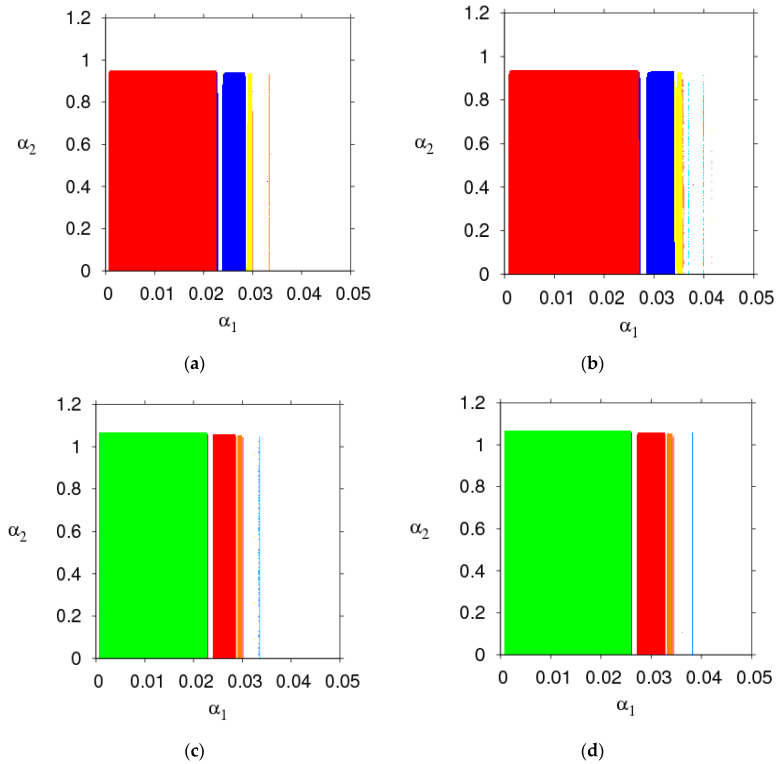
2D parameter bifurcation in the ($\alpha_{1},\;\alpha_{2}$) plane. (**a**) $\tau_{m}=0.5$; (**b**) $\tau_{m}=1$; (**c**) v=6; (**d**) v=7.

The parameter basin is a kind of two-dimensional bifurcation diagram, 2D bifurcation diagram of the System (9) in the ($\alpha_{1}$,$\;\alpha_{2})$ plane is displayed in Figure 3, which shows the route of the System (9) to chaos. 

Figure 3a,b shows the 2D bifurcation diagrams with the change of $\tau_{m}$. Different colors represent different periods, the stable region (red), 2-period (blue), 3-period (orange), 4-period (yellow), 5-period (green), 6-period (light blue), 7-period (purple), 8-period (coral), and the chaos (white). We can see the stable range of $\alpha_{1}$ changes from 0.0225 to 0.0271. By comparing the size of the red region, the stability region of the System (9) expands when the manufacturer recovery rate $\tau_{m}$ goes from 0.5 to 1.

Figure 3c,d shows the 2D bifurcation diagrams with v=6 and v=7; the stable region (green), 2-period (red), 3-period (blue), 4-period (orange), 5-period (yellow), 6-period (light blue), 7-period (purple), 8-period (coral), and chaos (white). In Figure 3c,d, the stable range of $\alpha_{1}$ changes from 0.0225 to 0.0267 and $\alpha_{2}$ is unchanged when v goes from 6 to 7. 

From Figure 3, we can conclude that increasing the retailer’s service value and manufacturer’s recycle rate expands the stability region of the Nash equilibrium point and make the market more competitive. Therefore, managers increase the appropriate product recovery rate and service is conducive to market stability.

### 4.2. The Influence of the Price Adjustment Speed on the System Behavior

**Figure 4:**
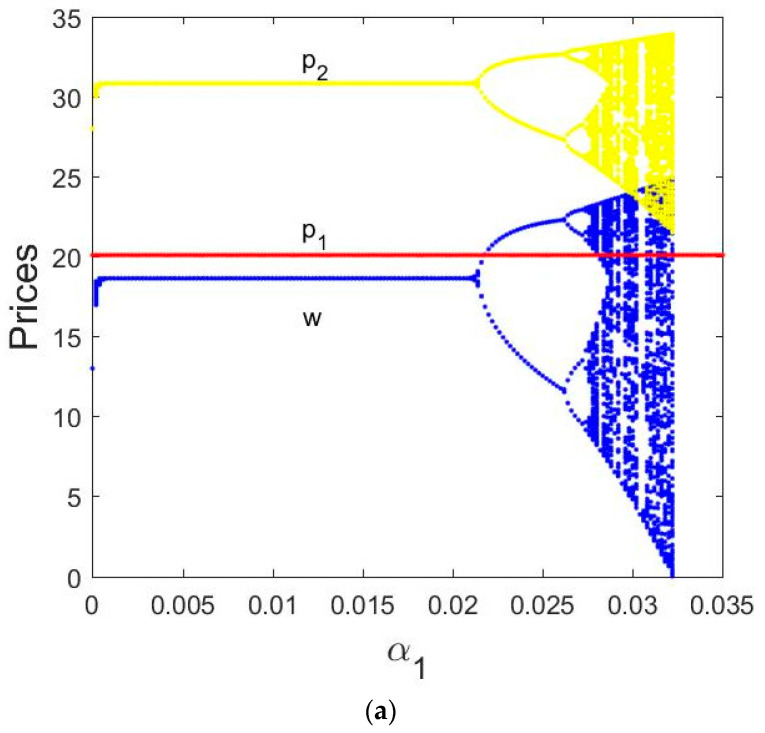

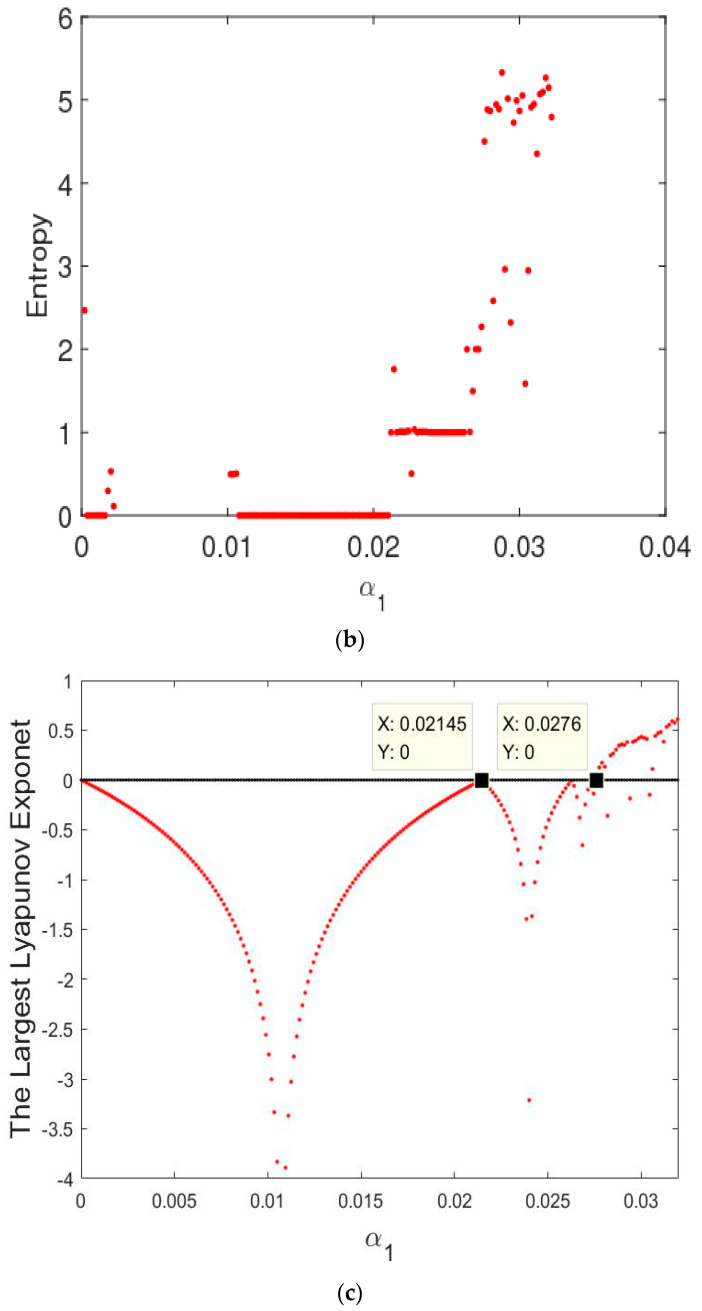
Bifurcation diagrams of the System (9) for $\alpha_{2}=0.01$ and $\alpha_{1}$ varying from 0 to 0.035. (**a**) Bifurcation diagram of prices in terms of $\alpha_{1}$; (**b**) the entropy diagram; (**c**) the corresponding largest Lyapunov exponents (LLEs) for $\alpha_{1}$ varying from 0 to 0.03.

Figure 4a shows the bifurcation diagram of the direct selling price, retail price, and wholesale price. We can see that the direct selling price $p_{1}$ is not affected by the change of $\alpha_{1}$. When $\alpha_{2}$ is fixed and the price adjustment parameter $\alpha_{1}\in \left(0,\;0.0215\right)$, the retail price $p_{2}$ and wholesale price w are fixed values. In the equilibrium state, the direct selling price $p_{1}=20.0842$, the retail price $p_{2}=30.8268$ and wholesale price w=18.6368; as $\alpha_{1}$ increases, the System (9) loses its stability and appears 2-period cycle bifurcation, then period-doubling bifurcation when $\alpha_{1}\in \left(0.0215,0.0276\right)$, and falls into chaos finally when $\alpha_{1}\in \left(0.0276,\;0.0325\right)$.

As can be seen from Figure 4b, when $\alpha_{1}\in \left(0.011,0.021\right)$, the entropy of the System (9) is equal to 0. At this time, the Nash equilibrium point is stable, the wholesale price w and retail price $p_{2}$ are fixed values, and the System (9) is in a stable state. However, $\alpha_{1}$ increases when $\alpha_{1}\in \left(0.022,0.0276\right)$, the entropy of the System (9), increases. The System (9) is in a doubly periodic bifurcation state, and the wholesale price w and retail price $p_{2}$ are unstable and appear multiple values. When $\alpha_{1}>0.027$, the entropy of the System (9) continues to increase, finally falls into chaos. 

Figure 4c shows the corresponding largest Lyapunov exponent (LLE) with $\alpha_{1}$ varying from 0 to 0.035, we can see that the LLEs is negative when $\alpha_{1}\in \left(0,\;0.0276\right)$, as $\alpha_{1}$ increases, the system enters bifurcation and period-doubling bifurcation states when the $\alpha_{1}=0.02145\;\mathrm{and}\;0.0276,$ it also means the LLEs equal to 0. When the LLEs are larger than 0, it indicates that the System (9) appears to be in a chaotic state.

**Figure 5:**
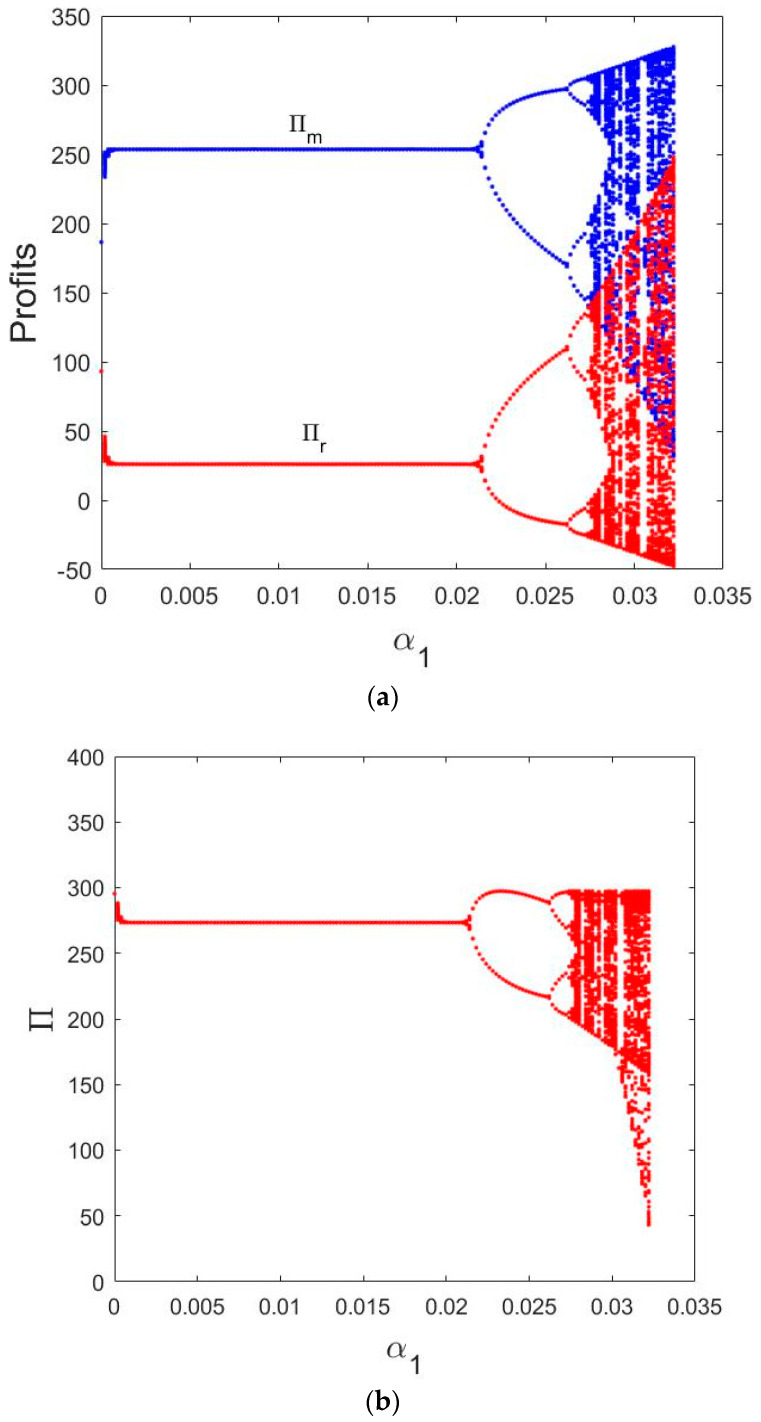
Bifurcation diagrams of the profits for $\alpha_{2}=0.01$ and $\alpha_{1}$ varying from 0 to 0.035. (**a**) Bifurcation diagram of profits in terms of $\alpha_{1}$; (**b**) Bifurcation diagram of total profit in terms of $\alpha_{1}$.

In Figure 5a, we can see that the trend of profit bifurcation graph of the manufacturer and retailer is similar to that of the price changes with the change of $\alpha_{1}$. The profits experience a period of stability when $\alpha_{1}\in \left(0,\;0.0215\right)$ and starts to double cycle bifurcation when $\alpha_{1}\in \left(0.0215,\;0.0276\right)$, finally enters a state of chaos.

Similarly, the total profit bifurcation diagram of the manufacturer and retailer is shown in Figure 5b, when $\alpha_{1}\in \left(0,\;0.0215\right)$, the total profit remains in a stable state. When $\alpha_{1}\in \left(0.0215,0.0276\right)$, the total profit starts to double cycle bifurcation and enters a state of chaos, finally.

From these figures, we can get that the larger price adjustment parameter will lead to the market into a state of chaos, while the smaller appropriate price adjustment is beneficial to the manufacturer and retailer’s profits to maintain the stability of the market.

**Figure 6:**
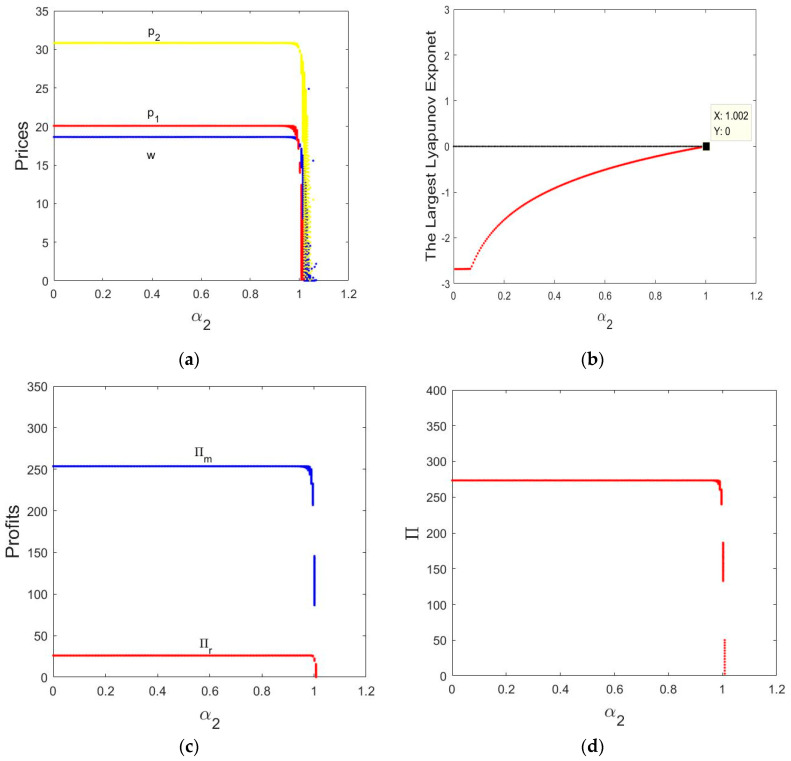
Wave shape chaos diagrams of the System (9) for $\alpha_{1}=0.01$ and $\alpha_{2}$ varying from 0 to 1.2. (**a**) Wave shape chaos diagrams of prices; (**b**) the corresponding LLEs in terms of $\alpha_{2};$ (**c**) the profits of the manufacturer and retailer in terms of $\alpha_{2}$; (**d**) total profit in terms of $\alpha_{2}$.

Figure 6a shows wave shape chaos diagrams of the System (9) for $\alpha_{1}=0.01$ and $\alpha_{2}$ varying from 0 to 1.2, we can see that the direct price $p_{1}$, retail price $p_{2}$ and wholesale price w remains stable state when the price adjustment parameter $\alpha_{2}\in \left(0,\;1.002\right)$. When $\alpha_{2}>1.002$, the manufacturer and retailer’s prices will move from a stable equilibrium to wave shape chaos. 

Figure 6b shows the LLEs with $\alpha_{2}$ varying from 0 to 1.2, the LLE is negative when $\alpha_{2}\in \left(0,\;1.002\right)$, it means that the System (9) is in a stable state. With the $\alpha_{2}$ increases, it indicates that the System (9) appears chaotic when the LLEs >0.

Figure 6c shows in an equilibrium state, the profits of the manufacturer and retailer are $\pi_{m}=249.3879$, $\pi_{r}=23.9805$, respectively. The profits of the manufacturer and retailer appear wave shape chaos as $\alpha_{2}$ increases.

The total profit of the manufacturer and retailer is 273.368 when $\alpha_{2}\in \left(0,\;1.002\right)$, as shown in Figure 6d. The total profit of the manufacturer and retailer falls into wave shape chaos when $\alpha_{2}>1.002$.

The above analysis shows that the manufacturer and retailer can obtain stable profits during the initial stabilization phase but as the price adjustment parameter $\alpha_{2}$ increases, the market will enter a chaotic state, and the manufacturer and retailer cannot plan for the long-term and achieve stable profits. Therefore, larger price adjustment parameters can make the system lose stability, a moderately small price adjustment speed is beneficial to the manufacturer and retailer.

### 4.3. The Influence of $v,\tau_{m}$ on the System Behavior

**Figure 7:**
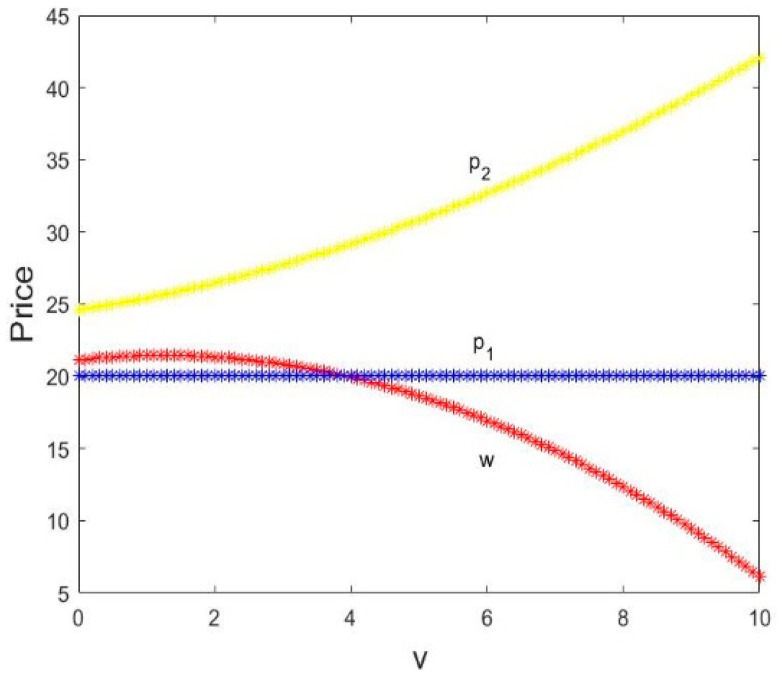
The changes in prices with v.

In Figure 7, the direct selling price $p_{1}$ is constant with v increase. The wholesale price w increases first and then decreases as the service value v increases and the retail prices $p_{2}$ increases with v increases. When v=4, the direct selling price $p_{1}$ is equal to the wholesale price w, and $p_{2}$ is greater than them. Therefore, increasing service value has nothing to do with the direct selling price, but can lead to the higher retail price and lower wholesale price.

**Figure 8:**
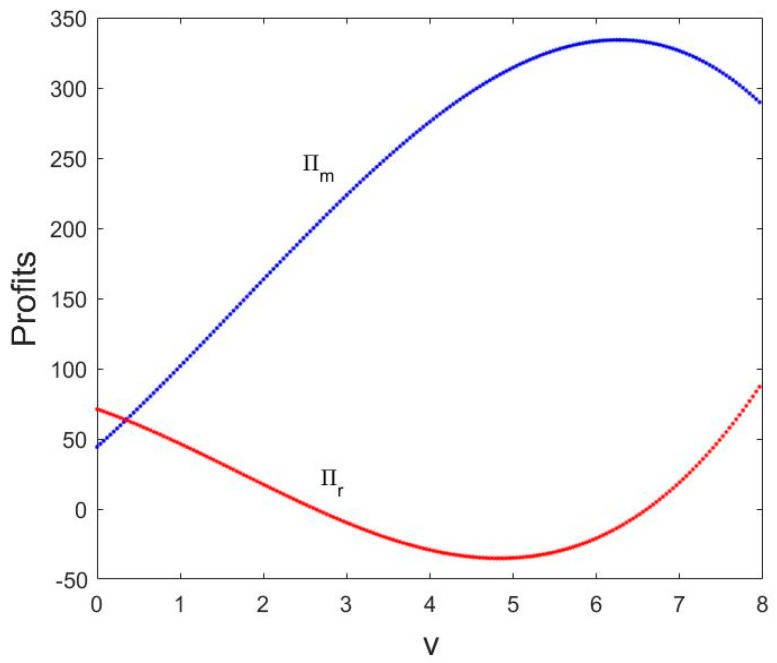
The changes in profits with v.

Figure 8 shows the change in profits with v changing when $\alpha_{1}=0.003,\;\alpha_{2}=0.01$. In Figure 8, we can see that in the equilibrium state, when v=0.46, the manufacturer and retailer’s profit is equal. Then, the manufacturer’s profit $\pi_{m}$ increases first and then decreases as the service value v increases, and the retailer’s profit $\pi_{r}$ first decreases until negative and then increases; the profit of the manufacturer is greater than that of the retailer when v increases. 

Thus, a hint to managers: increasing service input from retailers is beneficial to manufacturers. The retailer provides the appropriate service input to facilitate market competition. 

## 5. The Centralized Game Model

### 5.1. Model Construction

In the centralized decision model, both the manufacturer and the retailer aim to maximize profit, and they are cooperators of price decision. In this model, the retailer adjusts the retail price based on bounded rationality, and the manufacturer adjusts the direct selling price according to adaptability.

We take the first-order partial derivatives of $p_{1}$ and $p_{2}$ in π as follows:

$$\frac{\partial \pi }{\partial p_{1}}=\theta a-2b_{1}p_{1}+2kp_{2}+\left(b_{1}-k\right)\left(c_{1}-\tau_{m}\Delta \right)-kv-k\frac{\eta v^{2}}{2}$$

$$\frac{\partial \pi }{\partial p_{2}}=\left(1-\theta \right)a+2kp_{1}-2b_{2}p_{2}+\left(b_{2}-k\right)\left(c_{1}-\tau_{m}\Delta \right)+b_{2}v+\;b_{2}\frac{\eta v^{2}}{2}$$

Then, the Hesse matrix of the centralized decision model is as follows:

$$H^{c}=\left[\begin{array}{cc} -2b_{1} & 2k\\ 2k & -2b_{2} \end{array}\right]$$

Due to $-2b_{1}<0,-2b_{2}<0,$ so

$$\left|\begin{array}{cc} -2b_{1} & 2k\\ 2k & -2b_{2} \end{array}\right|=4b_{1}b_{2}-4k^{2}>0$$

The Hesse matrix is negative definite, so the total profit function is concave and only have the maximum solution, by solving $\frac{\partial \pi }{\partial p_{1}}=0$, $\frac{\partial \pi }{\partial p_{2}}=0$, we can get the optimal solution and the optimal total profit:

(12) $$p_{1}^{**}=\frac{\left(1-\theta \right)ak+\theta ab_{2}}{2\left(b_{1}b_{2}-2k^{2}\right)}+\frac{\left(c_{1}-\tau_{m}\Delta \right)}{2}$$

(13) $$p_{2}^{**}=\frac{\left(1-\theta \right)ab_{1}+\theta ak}{2\left(b_{1}b_{2}-2k^{2}\right)}+\frac{\left(c_{1}-\tau_{m}\Delta \right)}{2}+\frac{v}{2}+\frac{\eta v^{2}}{4}$$

$$\begin{array}{l} \pi^{**}=\left(p_{2}^{**}-\frac{\eta v^{2}}{2}-c_{1}+\tau_{m}\Delta \right)\left[\left(1-\theta \right)a-b_{2}p_{2}^{**}+b_{2}v+kp_{1}^{**}\right]+\left(p_{1}^{**}-c_{1}+\tau_{m}\Delta \right)(\theta a\\ \;-b_{1}p_{1}^{**}+kp_{2}^{**}-kv)-\frac{\xi \tau_{m}^{2}}{2} \end{array}$$

In the centralized decision model, the manufacturer and retailer make their next period price decision based on the marginal utility of the current period. When the current marginal utility is positive, the price will be raised in the next period; otherwise, the price will be reduced in the next period. The long-term price forecasting system is as follows:

(14) $$\{\begin{array}{l} p_{1}\left(t+1\right)=\mu_{1}p_{1}\left(t\right)+\left(1-\mu_{1}\right)\left(\frac{\left(1-\theta \right)ak+\theta ab_{2}}{2\left(b_{1}b_{2}-2k^{2}\right)}+\frac{\left(c_{1}-\tau_{m}\Delta \right)}{2}\right)\\ p_{2}\left(t+1\right)=p_{2}\left(t\right)+\mu_{2}p_{2}\left(t\right)\left[\left(1-\theta \right)a+2kp_{1}-2b_{2}p_{2}+\left(b_{2}-k\right)\left(c_{1}-\tau_{m}\Delta \right)+\;b_{2}v+\;b_{2}\frac{\eta v^{2}}{2}\right] \end{array}$$

where $\mu_{1}\;(0<\mu_{1}<1)$ is the adaptive adjustment parameter and $\mu_{2}>0$ is the adjustment parameters of finite rationality.

### 5.2. Model Analysis

We can obtain two equilibrium solutions by making $p_{1}\left(t+1\right)=\mu_{1}p_{1}\left(t\right)+\left(1-\mu_{1}\right))p_{1}^{**}$, $p_{2}\left(t+1\right)=p_{2}\left(t\right)+\mu_{2}p_{2}\left(t\right)\frac{\partial \pi }{\partial p_{2}}$:

$$E_{0}=\left(\begin{array}{cc} \frac{\left(1-\theta \right)ak+\theta ab_{2}}{2\left(b_{1}b_{2}-2k^{2}\right)}+\frac{\left(c_{1}-\tau_{m}\Delta \right)}{2}, & 0 \end{array}\right);$$

$$E_{1}=\left(\begin{array}{cc} \frac{\left(1-\theta \right)ak+\theta ab_{2}}{2\left(b_{1}b_{2}-2k^{2}\right)}+\frac{\left(c_{1}-\tau_{m}\Delta \right)}{2}, & \frac{\left(1-\theta \right)ab_{1}+\theta ak}{2\left(b_{1}b_{2}-2k^{2}\right)}+\frac{\left(c_{1}-\tau_{m}\Delta \right)}{2}+\frac{v}{2}+\frac{\eta v^{2}}{4} \end{array}\right).\;$$

The Jacobian matrix of the System (14) is given by:

$$J=\left(\begin{array}{cc} \mu_{1} & 0\\ 2k\mu_{2}p_{2} & 1+\mu_{2}\left[\left(1-\theta \right)a+2kp_{1}-4b_{2}p_{2}+M\right] \end{array}\right)$$

where $M=\left(b_{2}-k\right)\left(c_{1}-\tau_{m}\Delta \right)+\;b_{2}v+\;b_{2}\frac{\eta v^{2}}{2}$.

The stability of equilibrium points is determined by the eigenvalues of the Jacobian matrix evaluated at the corresponding equilibrium points. If we substitute $E_{0}$ and $E_{1}$ into the Jacobian matrix, we can get the following proposition.

**Proposition**: $E_{0}$*is boundary equilibrium points, $E_{1}$ is the Nash equilibrium solution.*

**Proof:** For equilibrium point $E_{0}$, the Jacobian matrix of System (14) is equal to:

$$J\left(E_{0}\right)=\left(\begin{array}{cc} \mu_{1} & 0\\ 0 & Y \end{array}\right)$$

where $Y=1+\mu_{2}\left[\left(1-\theta \right)a+2kp_{1}^{**}+\left(b_{2}-k\right)\left(c_{1}-\tau_{m}\Delta \right)+\;b_{2}v+\;b_{2}\frac{\eta v^{2}}{2}\right]$

Its eigenvalues at $E_{0}$ are as follows:

$$\lambda_{1}=\mu_{1};$$

$$\lambda_{2}=1+\mu_{2}\left[\left(1-\theta \right)a+2kp_{1}^{**}+\left(b_{2}-k\right)\left(c_{1}-\tau_{m}\Delta \right)+\;b_{2}v+\;b_{2}\frac{\eta v^{2}}{2}\right]$$

We can see that $\lambda_{1}<1,\;\lambda_{2}>1$ which indicates $E_{0}$ is an unstable point. More precisely, $E_{0}$ is the boundary equilibrium point. We can prove $E_{1}$ is the Nash equilibrium solution of the System (14).

Next, we analyze the stability of the equilibrium point $E_{1}$:

$$J\left(E_{1}\right)=\left(\begin{array}{cc} \mu_{1} & 0\\ 2k\mu_{2}p_{2}^{**} & 1+\mu_{2}B_{2} \end{array}\right)$$

where $B_{2}=\left(1-\theta \right)a+2kp_{1}^{**}-4b_{2}p_{2}^{**}+\left(b_{2}-k\right)\left(c_{1}-\tau_{m}\Delta \right)+\;b_{2}v+\;b_{2}\frac{\eta v^{2}}{2}$.

We consider the stability of the Nash equilibrium point, the corresponding characteristic polynomial of System (14) can be written as follows:

$$f\left(\lambda \right)=\lambda^{2}+U_{2}\lambda +U_{3}$$

where $U_{2}=1+\mu_{2}B_{2}+\mu_{1}$;

$U_{3}=\mu_{1}\left(1+\mu_{2}B_{2}\right)$;

$U_{2}$ and $U_{3}$ represent the trace and determinant of the Jacobian matrix J ($E_{1})$, respectively.

According to the Judging condition of the Jury’s equilibrium point stability, the necessary and sufficient conditions for obtaining the local stability of the Nash equilibrium point $E_{1}$ are:

(15) $$\{\begin{array}{l} \left(i\right):1+U_{2}+U_{3}>0\\ \left(ii\right):1-U_{2}+U_{3}>0\\ \left(iii\right):1-\left|U_{3}\right|>0 \end{array}$$

Solving the inequality Equation (15), we can obtain the stable region of the System (14). In the stable region, the System (14) is locally stable with the initial values of prices within a certain range. Because these limitations are very complex, solving the inequality Equation (15) is very complicated. Next, we give the stable region of System (14) through numerical simulation.

## 6. Numerical Simulations

In this section, we chose the same parameters as in the previous section and obtained $p_{1}=20.0842,p_{2}=28.6368$.

### 6.1. The Influence of $v\;and\;\tau_{m}$ on the Stability Region 

**Figure 9:**
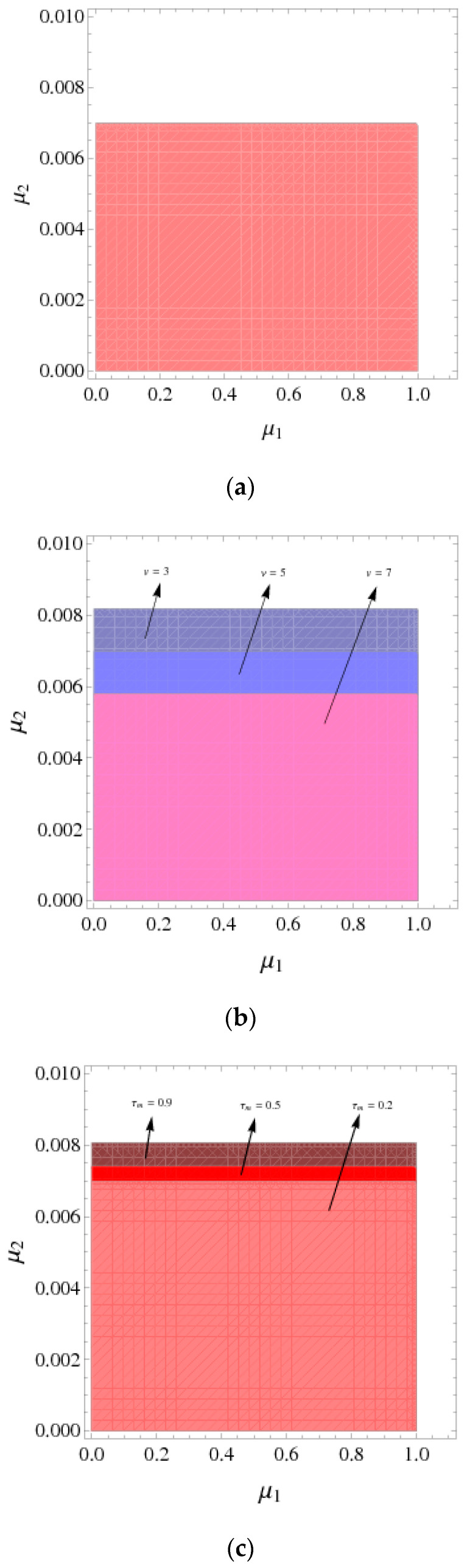
The stability regions of the System (14). (**a**) The stability regions of the System; (14) with $v=5,\tau_{m}=0.2;$ (**b**) the stability regions of the System (14) with different values of v; (**c**) the stability regions of the System (14) with different values of $\tau_{m}$.

Figure 9a shows the stability region of the System (14) with $v=5,\tau_{m}=0.2$. At this time, the stable range of the price adjustment parameter is that $\mu_{1}\in \left(0,\;1.002\right)$ and $\mu_{2}\in \left(0,\;0.00699\right)$, respectively.

In Figure 9b, when v=3, 5 and 7, the stability regions of the System (14) are represented by three different colors: pink, purple, and dark purple. We can see that when v=3, the stable range of $\mu_{2}$ is in [0, 0.0081] and the one of $\mu_{1}$ unchanged; when v=7, the stable range of $\mu_{2}$ is in [0, 0.0058] and the one of $\mu_{1}$ unchanged. It means that as the service value v increases, the stable range of $\mu_{2}$ decreases, the stable range of $\mu_{1}$ unchanged.

In Figure 9c, when $\;\tau_{m}=0.2,\;0.5,\;\mathrm{and}\;0.9,$ the stability regions of the System (14) are represented by three different colors: coral, red, and wine. When $\tau_{m}=0.5,$ the stable range of $\mu_{2}$ is in [0, 0.0074] and the one of $\mu_{1}$ unchanged; when $\tau_{m}=0.9,$ the stable range of $\mu_{2}$ is in [0, 0.0081] and the one of $\mu_{1}$ unchanged. It means the price adjustment parameters $\mu_{2}$ increases as the recovery rate $\tau_{m}$ increases.

According to the above analysis, if the manufacturer increases the recovery rate and the retailer reduces the service level, the stability region of the System (14) will be enlarged, which will make the market competition more intense.

**Figure 10:**
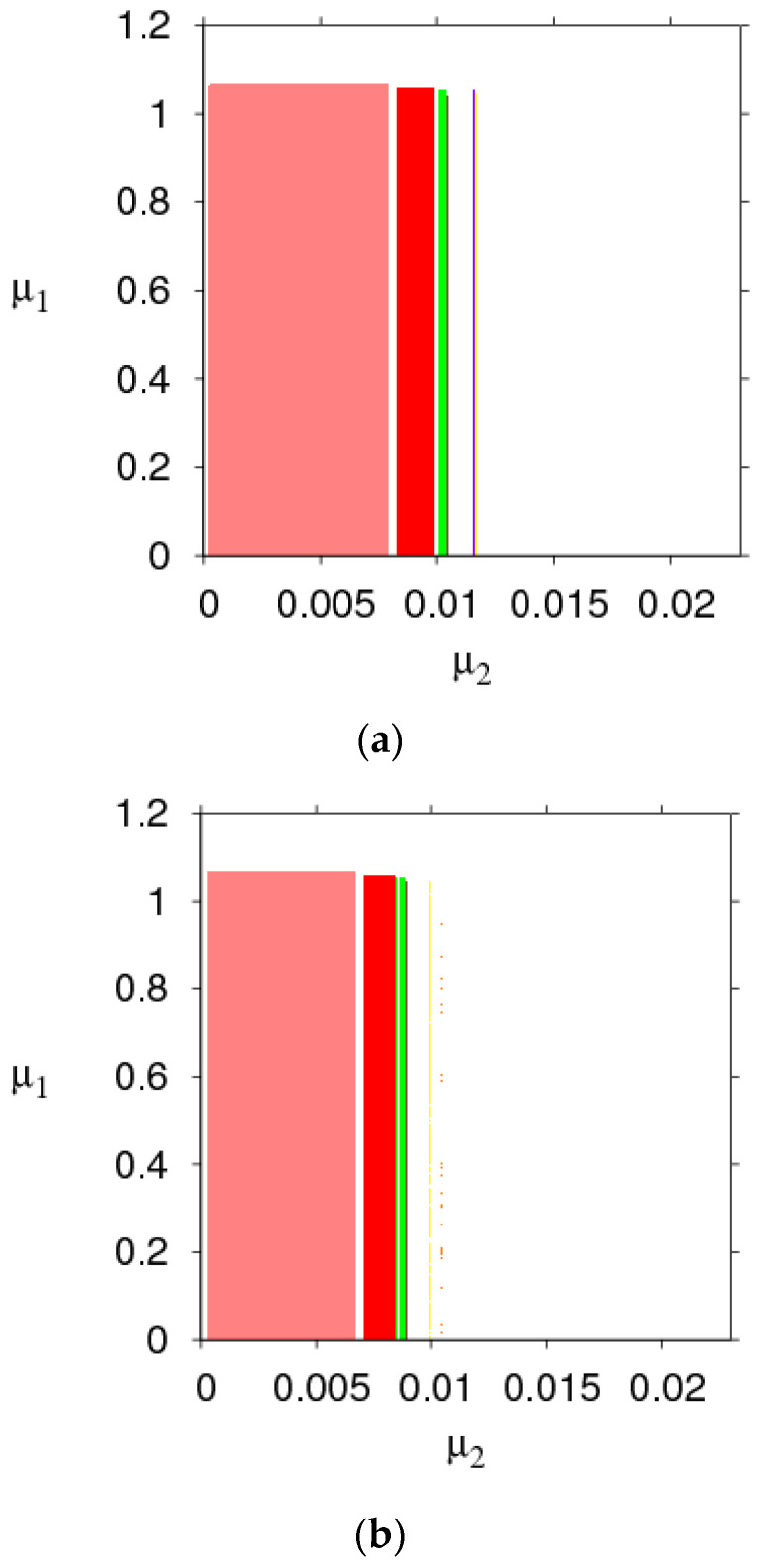

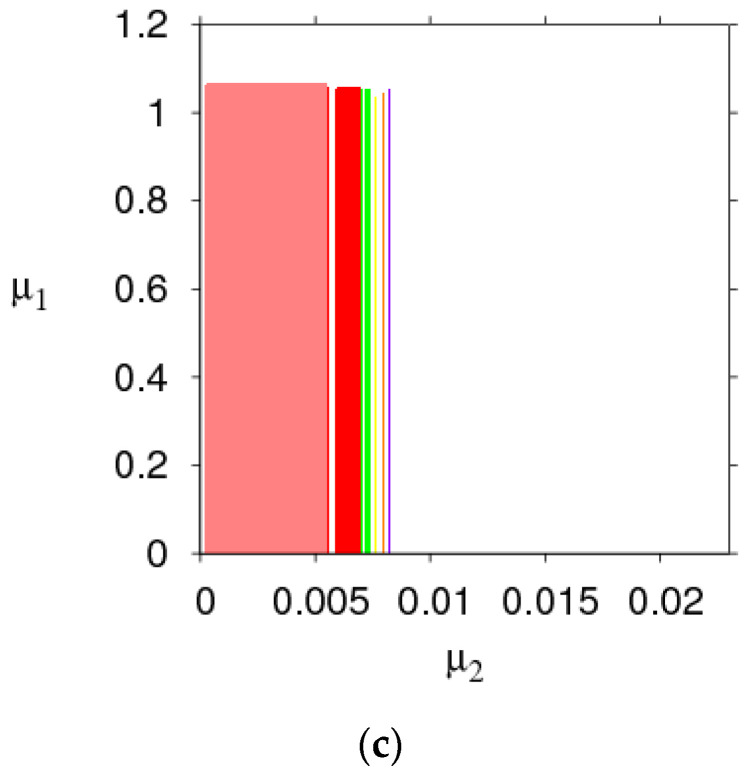
2D parameter bifurcation of the service value in the ($\mu_{1},\;\mu_{2}$) plane. (**a**) v=3; (**b**) v=5; (**c**) v=7.

Figure 10 is the 2D bifurcation diagram in planes of $\mu_{1}$ and $\mu_{2}$ with v=3, 5, and 7. Different colors represent different periods, the stable region (coral), period-2 (red), period-3 (green), period-4 (orange), period-5 (yellow), period-6 (blue), period-7 (purple), period-8 (red wine), and chaos (white). By comparing the size of the stability region (coral) in Figure 10 when v=3, the range of the coral region $\mu_{2}\in \left(0,\;0.0072\right)$, when v=5, the range of the coral region $\mu_{2}\in \left(0,0.0063\right)$ and when v=7, the range of the coral region $\mu_{2}\in \left(0,0.0058\right)$. We can obviously see that higher service values will shrink the stability region of the System (14).

**Figure 11:**
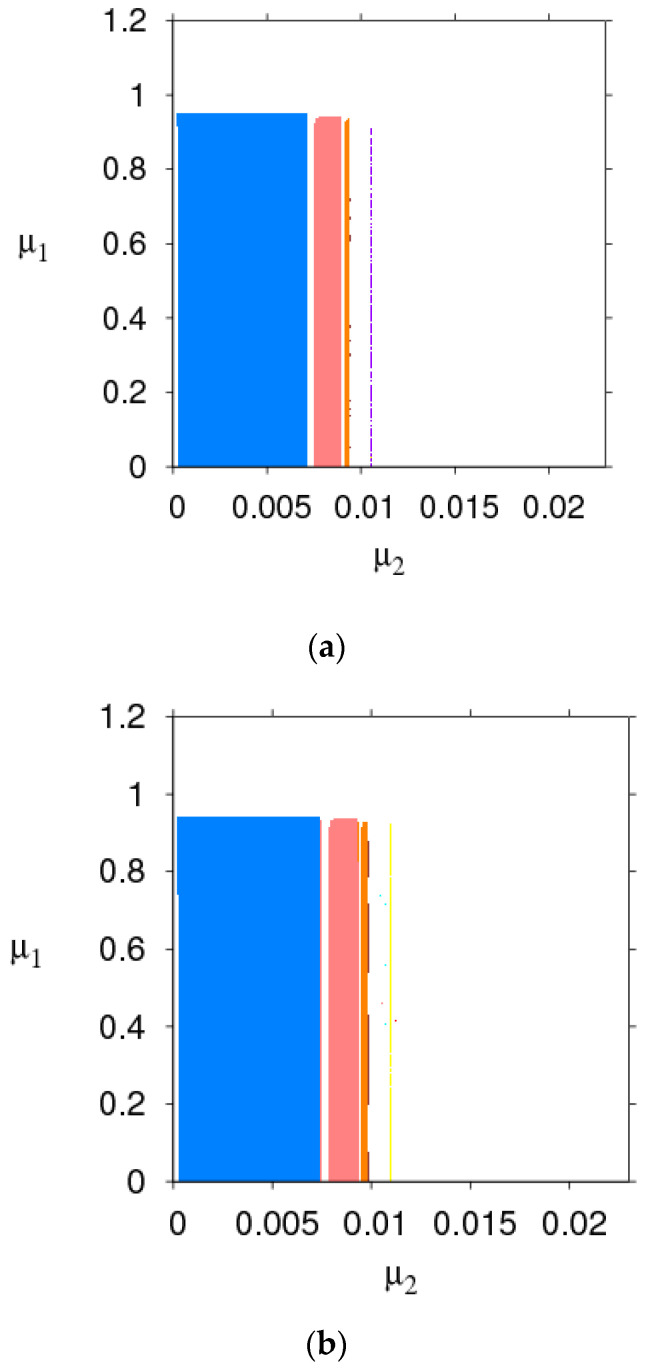

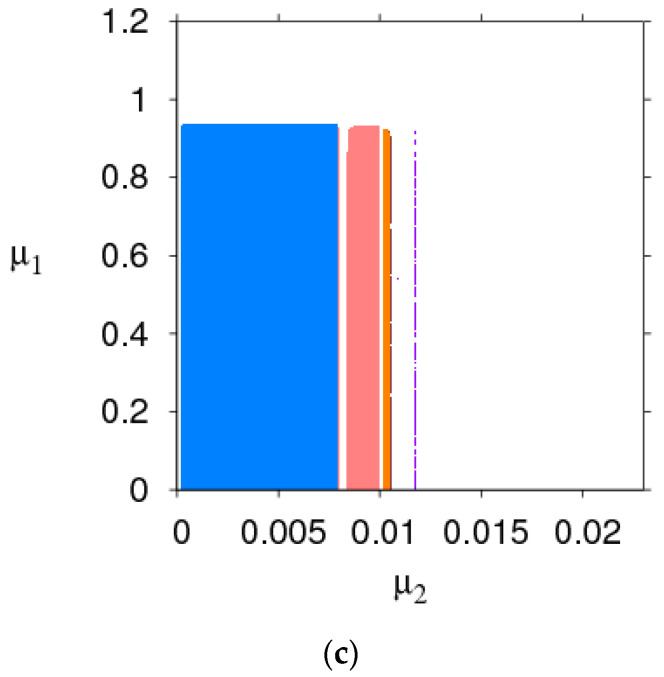
2D parameter bifurcation of recovery rate in the ($\mu_{1},\;\mu_{2}$) plane. (**a**) $\tau_{m}=0.5$; (**b**) $\tau_{m}=0.7$; (**c**) $\tau_{m}=1$.

Figure 11a–c is the 2D bifurcation diagram in planes of $\mu_{1}$ and $\mu_{2}$ with $\tau_{m}=0.5,\;0.7,\;\mathrm{and}\;0.9$. Different colors represent different periods, the stable region (blue), period-2 (coral), period-3 (yellow), period-4 (orange), period-5 (light blue), period-6 (green), period-7 (purple), period-8 (red wine), and chaos (white). By comparing the size of the stability (blue) region in Figure 11a–c, when $\tau_{m}=0.5$, the range of the blue region is $0<\mu_{2}<0.0068$. The range of the blue region is $0<\mu_{2}<0.0074$ when $\tau_{m}=0.7$ and the range of the blue region is $0<\mu_{2}<0.0081$ when $\tau_{m}=1$. We can see that the stability region of the System (14) will be expanded with $\tau_{m}$ increasing.

Through the above analysis, it can be obtained that appropriate higher the manufacturer’s recovery rate and smaller the retailer’s service value can expand the stable region of the System (14), which makes the market competition more intense.

### 6.2. The Influence of the Price Adjustment Speed on the Behavior of the System (14)

**Figure 12:**
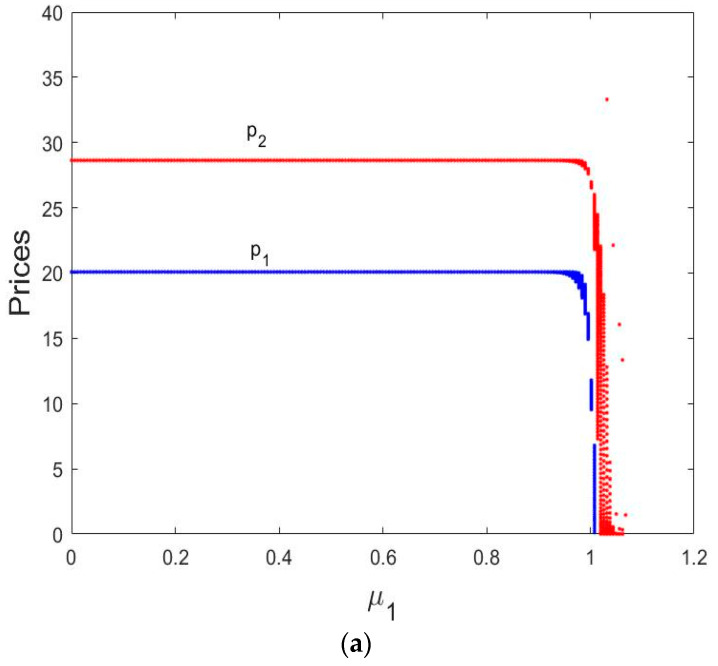

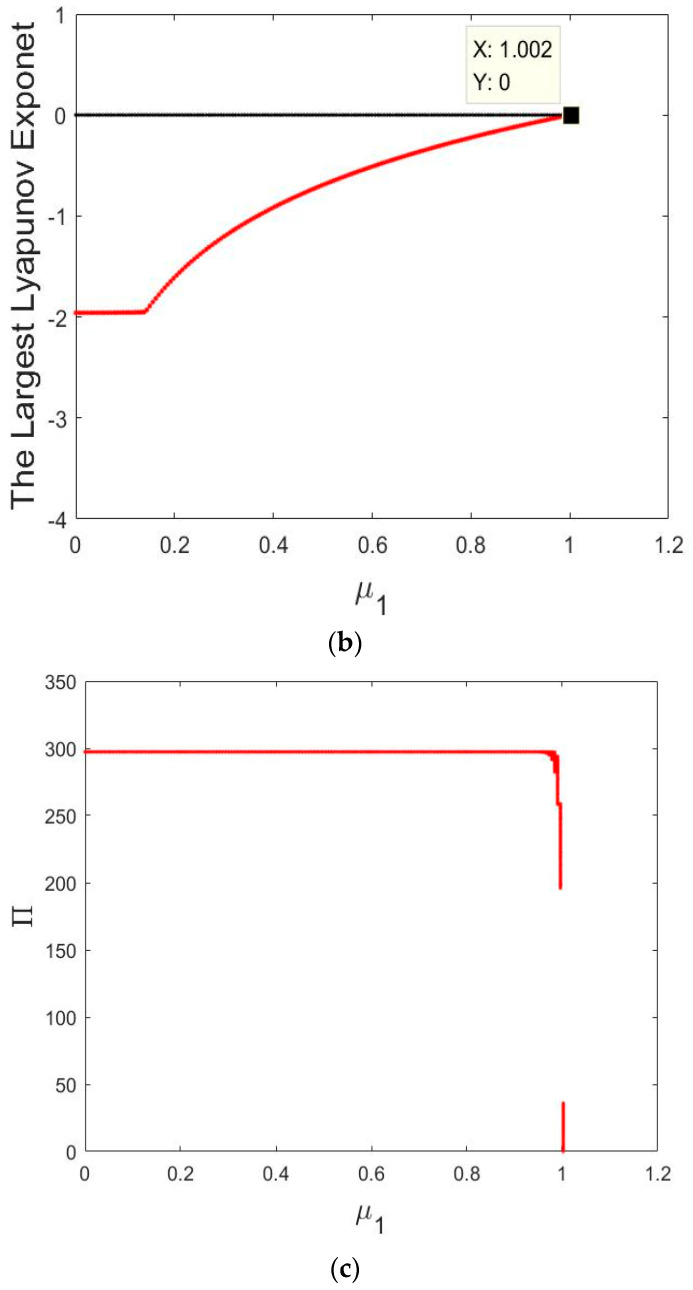
Wave shape chaos diagrams and the corresponding largest Lyapunov exponent of the System (14). (**a**) wave shape chaos diagram of prices in terms of $\mu_{1}$; (**b**) the corresponding largest Lyapunov exponent for $\mu_{1}$ varying from 0 to 1.2; (**c**) wave shape chaos diagram of the total profits in terms of $\mu_{1}$.

In Figure 12a, we can see that when $0<\mu_{1}<1.002$, $p_{1}$ and $p_{2}$ remains in a stable state. In the equilibrium state, $p_{1}=20.0842$ and $p_{2}=28.6368$. When $\mu_{1}>1.002$, the $p_{1}$ and $p_{2}$ move from a stable equilibrium to wave shape chaos.

Figure 12b shows the LLEs with $\mu_{1}$ varying from 0 to 1.2, the LLEs is negative when $0<\mu_{1}<1.002$; as $\mu_{1}$ increases, the LLEs is greater than 0, which indicates that the System (14) appears chaotic. 

Similarly, in Figure 12c, the total profit of the supply chain system remains in a stable state when $0<\mu_{1}<1.002$. In the equilibrium state, the total profit π=290.3489. When $\mu_{1}>1.002$, the System (14) appears in a wave shape chaos. 

**Figure 13:**
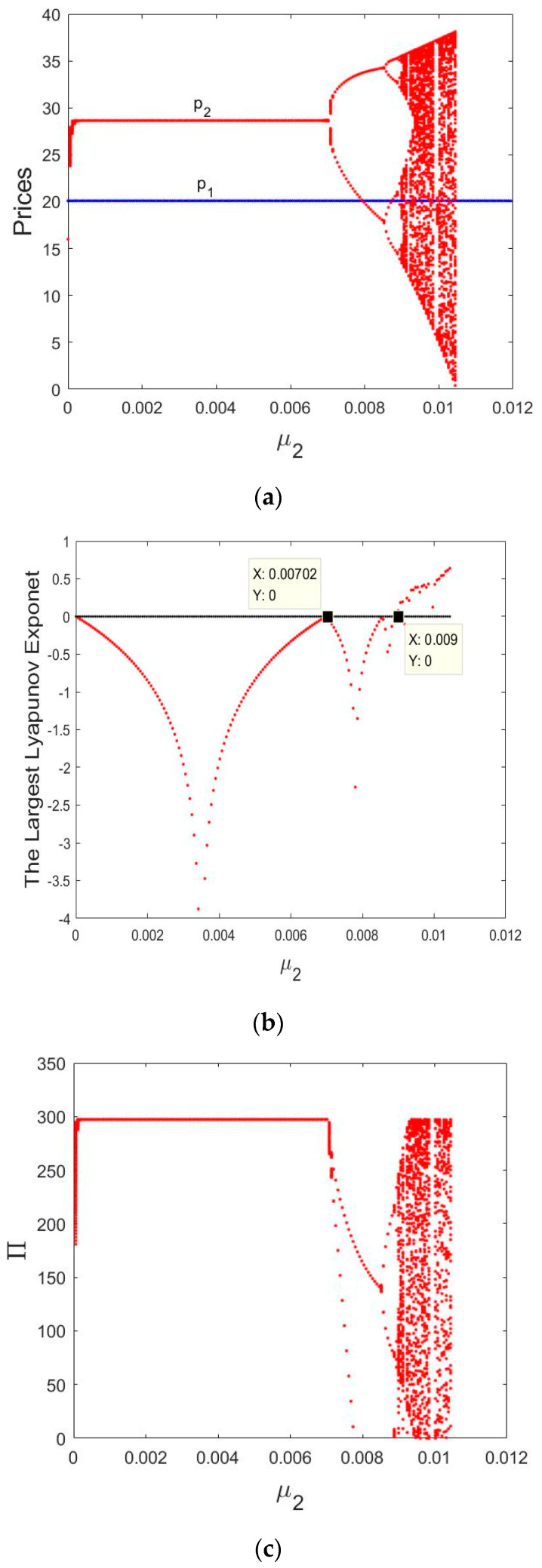
Bifurcation diagrams and the corresponding LLEs of the dynamic System (10). (**a**) Bifurcation diagram of prices in terms of $\mu_{2}$; (**b**) the corresponding largest Lyapunov exponent for $\mu_{2}$ varying from 0 to 0.012; (**c**) bifurcation diagram of the total profit in terms of $\mu_{2}$.

In Figure 13a, the $p_{1}$ is a fixed value with the change of $\mu_{2}$. When $\mu_{2}\in \left(0,\;0.00702\right)$, $p_{2}$ remains a stable state. As $\mu_{2}$ increases, $p_{2}$ loses stability and appears as a 2-period cycle bifurcation, then period-doubling bifurcation and then finally enters chaos. 

Figure 13b shows the corresponding LLEs for $\mu_{2}$ varying from 0 to 0.012, we can see that the LLEs is negative when $\mu_{2}\in \left(0,\;0.009\right)$. The System (14) appears bifurcation when $\mu_{2}=0.00702$ and $\mu_{2}=0.009$, respectively. When the LLEs  >0, it indicates the emergence of chaos.

Figure 13c is the bifurcation diagram of the total profit when $\mu_{1}=0.01$ and $\mu_{2}\in \left(0,\;0.012\right)$. Similarly, when $\;0<\;\mu_{2}<0.00702$, the total profit remains stability state. As $\mu_{2}$ increases, the total profit appears as a 2-period cycle bifurcation, then period-doubling bifurcation and then finally enters chaos.

Therefore, the manufacturer and retailer can obtain stable profits under the appropriate price adjustment speed, as the price adjustment parameter increases, the market will enter a chaotic state. After that, the retail prices of the retailer will fluctuate drastically. It will affect the manufacturer and retailer to make long-term plans and their own interests. So, smaller prices adjustment can benefit the market.

### 6.3. The Influence of $v\;and\;\tau_{m}$ on the System Behavior

**Figure 14:**
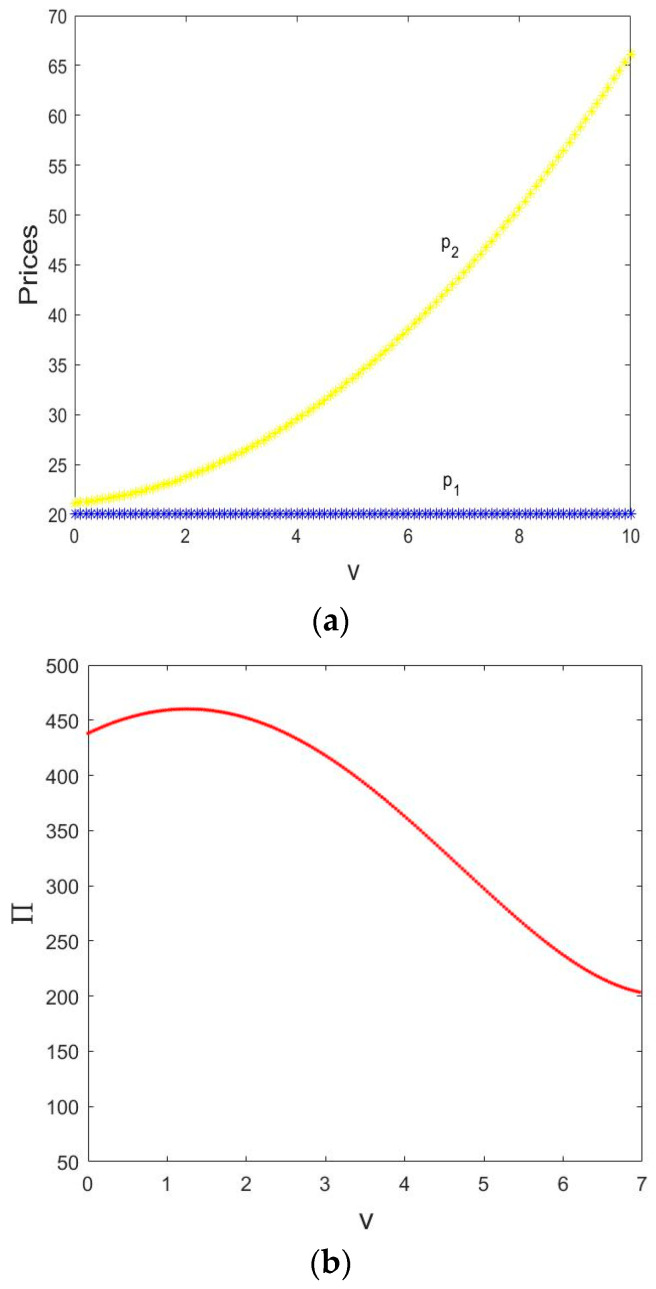

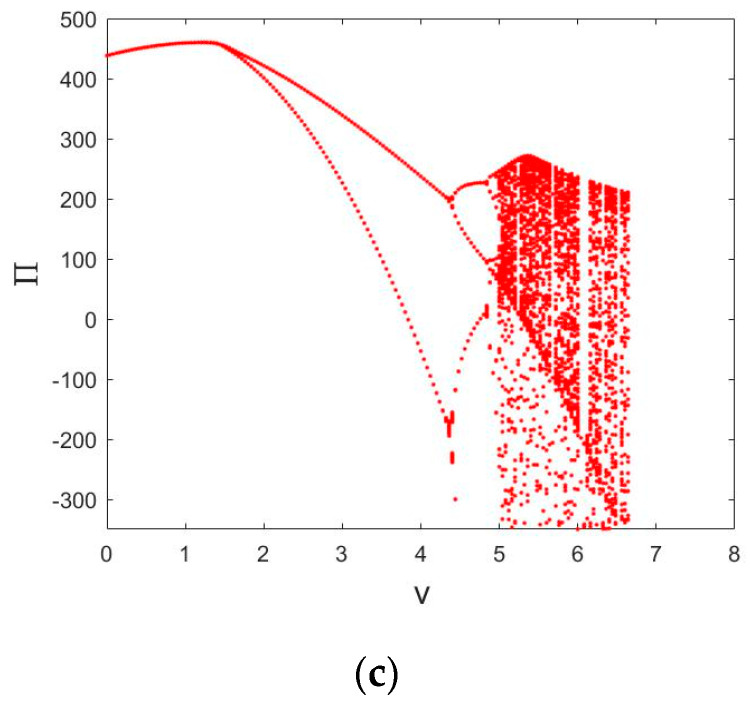
The Influence of $v\;and\;\tau_{m}$ on the System Behavior. (**a**) The change of prices with v; (**b**) the change of the total profit with v when $\mu_{1}=0.001,\;\mu_{2}=0.003;$ (**c**) the change of the total profit with v when $\mu_{1}=0.001,\;\mu_{2}=0.009$.

In Figure 14a, as the service value v increases, $p_{1}$ remains unchanged; $p_{2}$ gradually increases as v increases. Therefore, the service value increase can result in a higher retail price for the retailer and has no effect on the direct price.

Figure 14b shows the change of the total profit in an equilibrium state with $\mu_{1}=0.001,\mu_{2}=0.003$. As v increases, the total profit increases first and then decreases. When v=1.243, the total profit increased to the maximum.

In Figure 14c, we can see that the change of the total profit in the period-doubling bifurcation state with $\mu_{1}=0.001,\mu_{2}=0.009$. The total profit of the manufacturer and retailer increases when v∈(0, 1.34) and the total profit has the maximum value when v=1.34. The total profit appeared period-2 cycle bifurcation when v∈(1.34, 4.31). When v increases, the total profit appears period-doubling bifurcation, and enters a chaotic state finally. 

Therefore, we can know that an appropriate smaller service value can improve the total profit of the supply chain system, a larger service value can cause the market to enter a state of chaos. 

## 7. Conclusions

In this paper, we studied a dual-channel closed-loop supply chain in which a manufacturer considers waste product recovery and remanufacture, whereas a retailer considers the provision of services to customers. We build a Stackelberg game model and a centralized game model, study not only the static pricing strategy but also evolution characteristics of dynamic pricing of the two models. The profits of the manufacturer and retailer are described when parameters change. The following conclusions can be obtained.

(1) The stability region of the system expands in the Stackelberg game model, while shrinks in the centralized model as the retailer’s service value increases; the stability region of the system expands as the recovery rate of used products increases in the Stackelberg game model and the centralized game model. Therefore, the increase of product recovery rate by managers is more conducive to the stability of the system and the market competition.
(2) The system stability is significantly impacted by the price adjustment parameters; with large price adjustment parameter values, the system will bifurcate or even fall into chaos with a larger entropy value. Managers should adopt a relatively small rate of price adjustment to maintain the stability of the market.
(3) When the Stackelberg game model is in a stable state, the manufacturer’s profit increases first and then decreases, and the retailer’s profit first decreases until negative and then increases as the service value of the retailer increases. The total profit in the Stackelberg model is less than that in the centralized decision model. So the cooperation of the manufacturer and retailer is more conducive to maximizing system profit under the centralized decision.

Nonetheless, this paper has made several assumptions. Losing these assumptions may allow us to understand the interactive dynamics of the model better. For instance, low-carbon behaviors of customers in the closed-loop supply chain will be considered and the model will close to the actual situation. Second, sales promotion activity should be taken into account, as it may shed lights on whether the current results will hold. These problems will be investigated in our future research.

## Figures and Tables

**Table 1:** Symbols used in this paper and their meanings.

	The Potential Market Size
a	The basic demand of market
θ	The customer’s loyalty to the traditional retailer channel, θ∈(0, 1)
$b_{1}$	The price elasticity coefficient of the direct channel
$b_{2}$	The price elasticity coefficient of the traditional channel
k	The cross-price sensitivity between two channels $\left(k<b_{1},k<b_{2}\right)$
v	Service value
η	The service cost parameters of the traditional channel
$\tau_{m}$	The recovery rate of waste products
ξ	The recovery cost coefficient of the manufacturer
$c_{1}$	The unit production cost of a new product
$c_{2}$	The unit production cost of a remanufactured product ($c_{1}>c_{2})$
w	The wholesale price for the retailer
$p_{1}$	The direct selling price of the manufacturer
$p_{2}$	The retail price of the retailer
